# A Unique Case of Chylothorax Occurring Post-Chest Tube Insertion

**DOI:** 10.7759/cureus.41999

**Published:** 2023-07-17

**Authors:** Faisal Syed, Qasim Khurshid, Felix Wireko, Vishal Poddar

**Affiliations:** 1 Internal Medicine, Howard University Hospital, Washington DC, USA; 2 Pulmonary and Critical Care, Howard University Hospital, Washington DC, USA

**Keywords:** hydropneumothorax, emboliztion, chest tube, pleural effusion, chylothorax

## Abstract

Chylothorax is a relatively rare condition characterized by the accumulation of chyle, a milky lymphatic fluid, within the pleural space. It occurs because of disruption or obstruction of the thoracic duct or its tributaries, leading to chyle leakage into the pleural cavity. We present an interesting case of chylothorax that occurred as a complication post-chest tube insertion. A 66-year-old patient presented with hypotension and shortness of breath. Initial chest X-ray in the emergency room showed a right-sided hydropneumothorax requiring chest tube placement. Later on, the patient was transferred to the medical intensive care unit for respiratory failure. Chest tube drainage was initially serosanguineous but later changed to milky-white drainage. Pleural fluid analysis showed a triglyceride level of 208, confirming chylothorax. Conservative treatment was initiated with a low-fat diet and octreotide. The plan was to schedule the patient for thoracic duct embolization in view of continuous chylous drainage, but due to family preference, the procedure was deferred. This case report provides an overview of chylothorax, including etiologies and diagnostic options, and shows the importance of taking a multidisciplinary approach to finalize management strategies.

## Introduction

Chylothorax occurs when there is chylous fluid present in the pleural space. The thoracic duct is the main duct that helps transport chyle and eventually drains into the internal jugular vein. Chyle contains a large number of triglycerides. Causes of chylothorax can be traumatic or nontraumatic. Nontraumatic causes include malignancies, sarcoidosis, and congenital lymphatic duct malformations [1]. Here we present the case of a patient who came in because of hypotension and was found to have a right-sided hydropneumothorax. A chest tube was placed and initially was draining serosanguineous fluid but later changed to milky white chylous fluid. The goal of this case report is to learn about chylothorax secondary to trauma, identify chylothorax through lab work, and outline treatment plans such as conservative management, pleurodesis, and thoracic duct embolization.

## Case presentation

**Figure 1:**
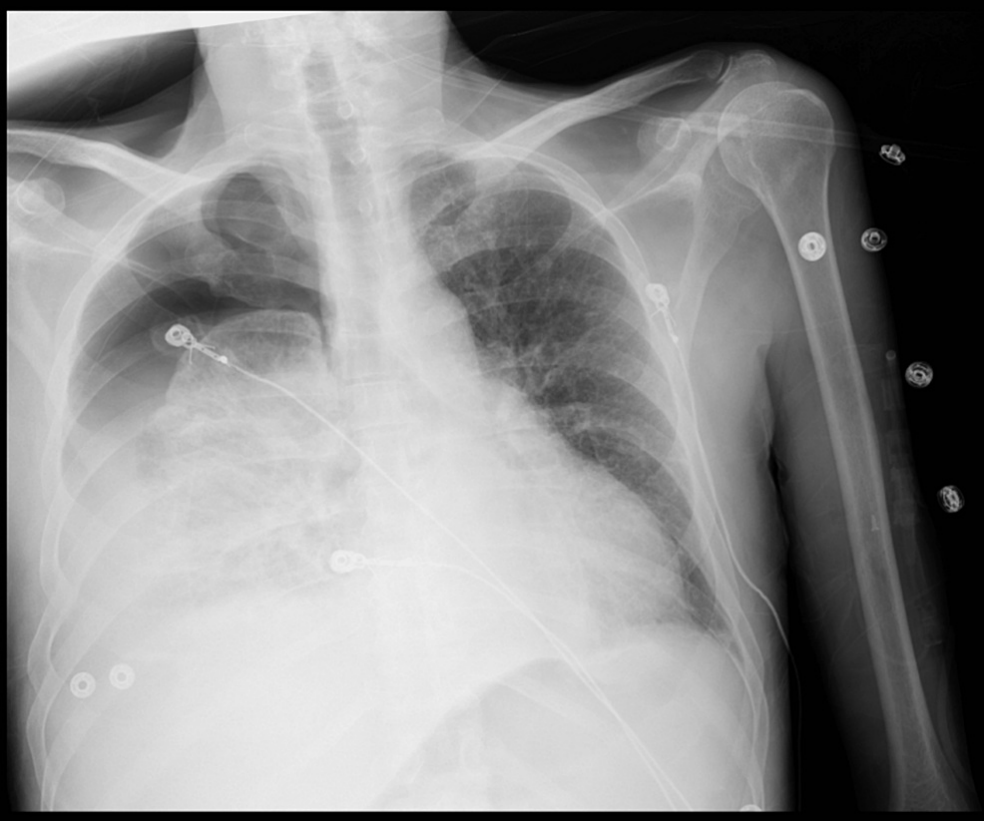
Initial chest X-ray showing right-sided hydropneumothorax

A 66-year-old male with a past medical history of hypertension, esophagitis with esophageal strictures secondary to Helicobacter pylori needing prolonged G-tube, and chronic hepatitis C infection was sent from a nursing home because of profound hypotension and shortness of breath. Initial vitals showed a BP of 57/33mmHg, a pulse of 85/minute, a temperature of 97.2°F, and an oxygen saturation of 96% in room air. While in the emergency room, a chest X-ray showed a right hydropneumothorax with a left mediastinal shift (Figure 1).

**Figure 2:**
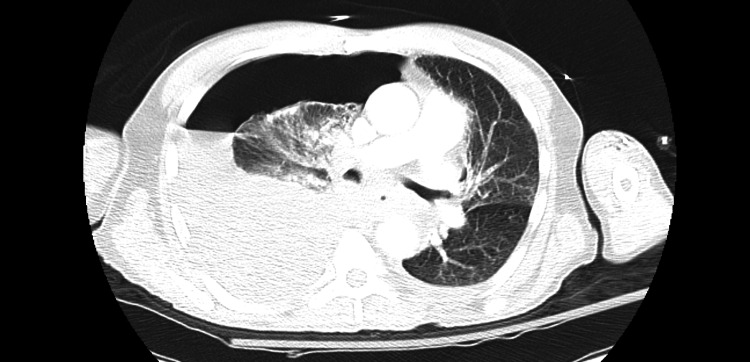
CT angiogram of the chest showing right-sided pleural effusion

CT angiogram of the chest confirmed right-sided pleural effusion with hydropneumothorax (Figure 2). 

**Figure 3:**
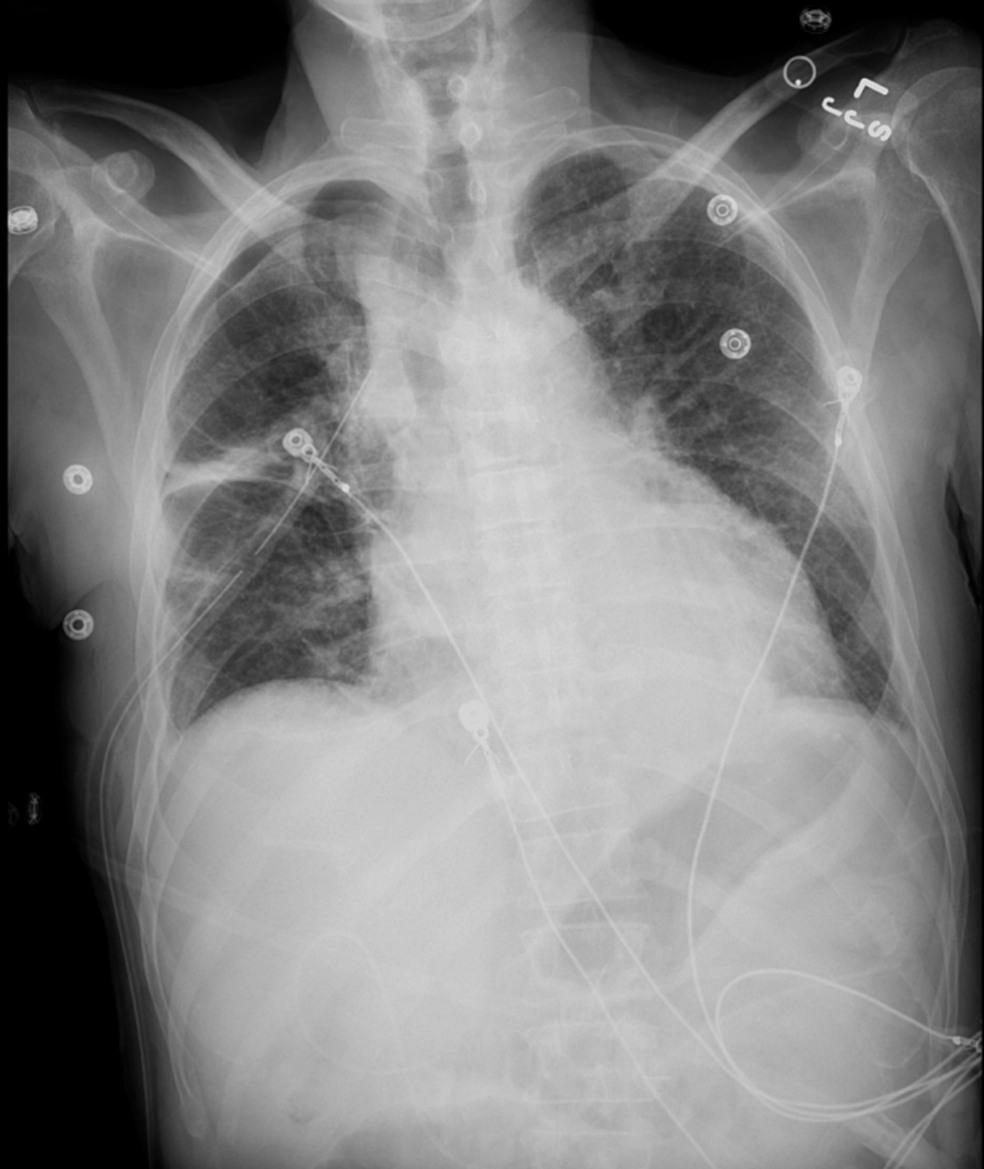
Chest X-ray post right-sided chest tube insertion

**Figure 4:**
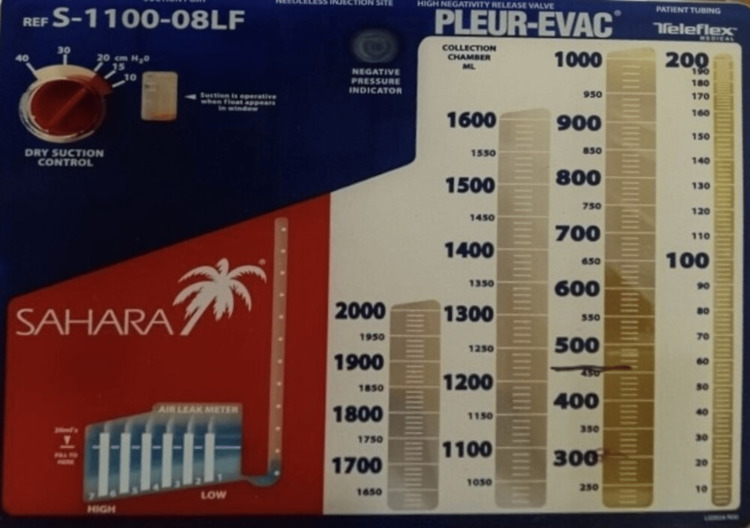
Collection chamber showing milky white chylous fluid

He was admitted to the medicine service for further management. Cardiothoracic surgery was consulted, and a right-sided chest tube was placed (Figure 3) for continuous drainage, with the pleural fluid being sent for first analysis. He was later transferred to a medical intensive care unit (MICU) for acute hypoxic respiratory failure, eventually requiring intubation. The patient’s temperature continued to spike, and a second set of pleural studies were sent. Pleural fluid culture and blood culture revealed Klebsiella pneumoniae. The patient was continued on antibiotics, and the chest tube continued to drain 800-1500ml fluid daily. Drainage was initially serosanguineous but later became chylous (Figure 4).

Due to this change, a third set of pleural fluid analyses was sent to measure the triglyceride level, which came back at 208, confirming chylothorax (Table 1).

**Table 1:** All three pleural analyses were done during the hospital course, with the third analysis showing a triglyceride level of 208 LDH: lactate dehydrogenase; RBC: red blood cell; pH: potential of hydrogen

	First pleural fluid analysis	Second pleural fluid analysis	Third pleural fluid analysis
Body fluid glucose	205	49	134
Body fluid LDH	542 (high)	1586 (high)	490 (high)
Body fluid total protein	<3.0	<3.0	<3.0
Body fluid triglyceride	-	-	208
Body fluid appearance	Turbid	Turbid	Cloudy
Body fluid color	Brown	Light yellow	Orange
Body fluid RBC	17000 (high)	36000 (high)	9000 (high)
Body fluid lymphocytes	40	28	18
Body fluid pH	7.5	6.5	-
Body fluid total nucleated cells	25041	53547	975
Body fluid neutrophil	60	61	67

**Figure 5:**
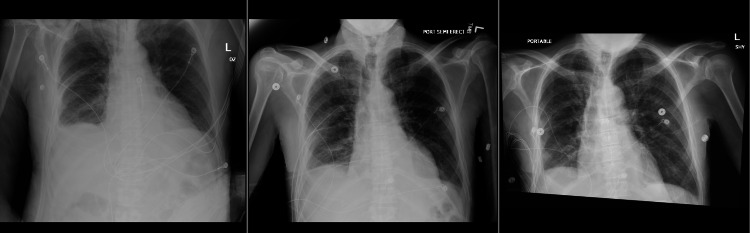
Serial chest X-ray post-chest tube removal showing improvement in pleural effusion (left to right)

The patient was then placed on a low-fat diet with octreotide 50mcg three times a day (TID) and was then transferred back to the medicine floor after successful extubation and clinical improvement. The chest tube was in place and continued to drain chylous fluid. This required a multidisciplinary approach involving the cardiothoracic team, interventional radiology, and internal medicine. A decision was made to do a lymphangiogram with embolization, but due to the patient’s age, the family preferred conservative management. The chest tube was later removed, and serial chest X-rays post-chest tube removal showed significant improvement in right-sided effusion (Figure 5). The patient remained clinically stable and was transferred back to the nursing facility.

## Discussion

Chylothorax is an accumulation of chyle in pleural fluid due to disruption of thoracic duct flow in the chest. It is a rare complication seen in malignancies and can also be seen in postsurgical procedures involving cardiac structures [2]. The course of the thoracic duct originates in the cisterna chyli around the second lumbar vertebra, passes through the aortic hiatus and the posterior mediastinum, and eventually terminates in the internal jugular or subclavian vein [3]. Traumatic chylothorax is usually iatrogenic in etiology and occurs post-surgery. Although rare, blunt trauma associated with fractures can also cause chylothorax [4].

Chylothorax as a complication of post-chest tube insertion is very rare. It is usually a late manifestation and can occur up to seven days after chest tube insertion. It should especially be considered in cases where other nontraumatic causes have been ruled out [5].

The thoracic duct anatomy determines whether the chylothorax location is on the right or left side. The thoracic duct crosses the mediastinum around the fifth thoracic vertebra; hence, any lymphatic injury below this level causes right-sided effusion. Inversely, anything above this level will cause left-sided effusion. The depth of chest tube insertion ranges from 5 to 15cm, and its predetermination can minimize the risk of this complication [6].

Chylothorax can be diagnosed by measuring triglyceride levels in pleural fluid analysis. Levels greater than 110 mg/dl are highly suggestive of chylous effusion. Levels < 50mg/dl exclude chylothorax. In equivocal cases, measurement of cholesterol levels can also be helpful, especially when cholesterol crystals are seen in microscopy [7]. It is essential to rule out other nontraumatic causes; there were no signs of lymphoma or any other malignancy in our case.

Chylothorax is initially managed conservatively. Options include dietary modifications, octreotide, and repeating thoracentesis. Octreotide works by decreasing intestinal motility and overall chyle formation. A low-fat diet helps reduce the formation of lymph. Total parenteral nutrition (TPN) is an alternative if diet modifications fail to bring about any changes. If conservative measures fail, pleurodesis has been shown to treat chylothorax when output is low. Some refractory cases can be treated with the transhepatic intrajugular portosystemic shunt (TIPS) procedure [8].

Thoracic duct embolization is also an alternative to consider when treating chylous leaks. Embolization has been shown to have a high success rate with minimal complications [9]. A study by Itkin et al. reported the outcomes of thoracic duct embolization in 109 patients with chylous leaks. These leaks were caused by traumatic damage to the thoracic duct. Catheterization of the thoracic duct was achieved in 73 patients (67%). In 71 of these 73 patients, embolization of the thoracic duct was performed. Resolution of the chyle leak was observed in 64 of 71 patients (90%) post-embolization [10].

## Conclusions

Chylothorax is a complex condition that requires a comprehensive understanding of its etiology, clinical presentation, and management strategies. Chylothorax is an infrequent complication post-chest tube insertion. Our case report documents a patient who came because of a right hydropneumothorax requiring chest tube placement and who developed chylothorax a few days later. Traumatic causes of chylothorax should always be considered in patients undergoing thoracic intervention, especially when other causes like lymphoma or malignancy have been ruled out. Chest tube insertion, surgical manipulations, and central venous catheter insertion are some interventions that can cause chylothorax. The critical point in diagnosing chylothorax through lab work will be checking triglyceride levels in the pleural fluids. Management consists of maintaining a low-fat diet and using octreotide. In our case, the medical team made arrangements for thoracic duct embolization, but the patient’s family later refused the procedure. Although the patient responded to dietary changes and octreotide, it is essential to remember that thoracic duct embolization is valuable and safe in treating traumatic chylothorax and can be considered a first-line option.
